# Optimizing EFL learning: exploring the role of learner background factors and the nuances of their effects on intrinsic and extrinsic motivation among university students in a mixed-methods study

**DOI:** 10.1186/s40359-024-02034-8

**Published:** 2024-10-05

**Authors:** Sara Kashefian-Naeeini, Nasrin Shokrpour, Farhad Pakdel

**Affiliations:** Department of English Language, School of Paramedical Sciences, Sara Kashefian-Naeeini, Shiraz University of Medical Sciences, Shiraz, Iran

**Keywords:** Intrinsic motivation, Extrinsic motivation, Learner variables, Mixed-methods study

## Abstract

**Introduction:**

Motivation is a driving force behind man’s behavior which has led to many psychological studies throughout the world. Moreover, it is the fuel for successful learning. While intrinsic motives provide the internal rewards, extrinsic motivation supplies the required external rewards to keep the engine of learning running. Knowing the factors which impact intrinsic/extrinsic motivation helps educators target their efforts at a higher level and make more informed decisions.

**Method:**

This study intends to examine how intrinsic vs. extrinsic motivation may be influenced by demographics including learners’ background factors of major, age, and occupation and to determine the relationships that may exist between intrinsic and extrinsic motivation. To this end, an explanatory sequential mixed-methods research was conducted at one of the public universities in Shiraz on the majors of Elementary Education, Educational Affairs, Social Studies and Theology, and the Arabic language which were selected through cluster sampling. These students were having their English courses at the university. Based on Krejcie and Morgan’s formula for sample size, a questionnaire was administered to 100 participants to collect quantitative data. Moreover, semi-structured interview sessions were conducted with one fourth of the participants.

**Results:**

Using Multiple Analysis of Variance (MANOVA) and Pearson correlation, we found that learners’ background factors of age, field of study and professional status did not affect intrinsic nor extrinsic motivation. However, significant and positive relationships were found between intrinsic motivation and total motivation index, and between extrinsic motivation and total motivation index. Qualitative data obtained from the interviews were analyzed using a thematic analysis. The results of the interviews showed some new illuminating trends as revealed from the participants’ responses and it was found that most interviewees followed intrinsic motives and considered motivation as a factor of great significance.

**Conclusion:**

In the English as a Foreign Language (EFL) context in which our study was conducted, instructors can follow more similar classroom motivational techniques and strategies since neither type of motivation was influenced by the variables of different major, age and occupation. Though many studies have shown that EFL learners are more extrinsically motivated in comparison with ESL ones, our study revealed that participants were more intrinsically motivated. Thus, this study may be replicated in other educational contexts such as an ESL context. The study can also be repeated in some universities in which other educational systems such as coed education is used to see the possible similarities and differences. Motivation is the important stimulant to impel the learners to achieve their learning goals; thus, it should receive sufficient attention in various educational settings.

**Supplementary Information:**

The online version contains supplementary material available at 10.1186/s40359-024-02034-8.

## Introduction

Motivation is a psychological process which provides goal and direction for human behavior and supplies the necessary impetus to keep the engine of learning running. Since motivation is considered as a significant determinant of academic success [1] which plays a key role in successful language learning, second/foreign language motivation has been the target of many studies.

Students’ success can be influenced by various factors, and motivation, as a multifaceted variable, is definitely one of the key contributors. One of the most common motivational orientation types are the intrinsic and extrinsic motivational orientations which have been used in the present study. Intrinsic motivation is the self-desire that urges a student to participate in an activity and helps him/her to engage in learning for the sake of learning. Intrinsic motivation emanates from within a learner, characterized by a genuine desire to learn for the sake of learning itself. A student driven by intrinsic motivation participates in learning due to the pleasure and satisfaction it brings [2]. This form of motivation serves as a pathway to finding fulfillment from within oneself. On the other hand, extrinsic motivation leads a student to pursue learning in anticipation of receiving praise, good grades, and external rewards. This motivational approach involves incentives originating from external sources [3], and it is driven by external factors outside the individual. The interplay between these types of motivation is crucial for grasping how students approach their educational experiences. Therefore, an individual’s total motivation is a combination of intrinsic and extrinsic motivation. Intrinsic and extrinsic motivation may be influenced by some factors. These factors include a number of personality traits and learner background variables.

The role of motivation in the learning process has been deemed significant for boosting learner engagement and accelerating positive results. In a world where English acts as a lingua franca, it is vital to grasp the complexities of motivation in EFL environments. Past research has shed light on the intrinsic and extrinsic aspects of motivation, emphasizing their significance in academic performance and overall language acquisition. Nonetheless, the interaction between these motivational types and various learner background factors such as age, field of study, and professional status has not been thoroughly investigated.

The way learner background factors interact to impact intrinsic versus extrinsic motivation is crucial not only for educators and practitioners of education but also for policymakers in designing suitable learning interventions. By comprehending these connections, instructional strategies will be tuned to address the motivational requirements of various learners. Thus, one important gap in the existing literature is the minimal exploration of how learner background factors shape motivational orientations. Moreover, the interplay between multiple factors remains unexplored. The absence of all-inclusive mixed method studies examining among different learner background factors and their effect on intrinsic vs. extrinsic motivation is another obvious research gap. While current research has already concentrated on individual factors such as age, gender and cultural background on motivation, the need of studies which consider different factors within a mixed method framework is deeply felt. Moreover, a large number of studies have been targeted to determine the effects of factors such as professional status on intrinsic vs. extrinsic motivation in work settings; however, these effects and interplay of factors such as professional status or age need to be examined in education settings, too.

Existing research focuses on either intrinsic or extrinsic motivation and scrutinizes the factors which may separately affect one of the aforementioned motivational types; however, these two types of motivation may interact and affect each other. Likewise, most studies lean towards quantitative methodologies using only limited questionnaires that do not capture the rich nuanced experience of learners, whereas a mixed method study which combines quantitative and qualitative methods can offer a more holistic picture of intricacies and relationships among variables and clarify how different learner background factors of age, major and professional status may influence motivation and provide a better comprehension of and a deeper insight into the core dynamics that navigate intrinsic and extrinsic motivation. Therefore, the present study expands the existing understanding of the area of motivation in general and intrinsic/extrinsic motivation in particular.

Motivation plays a key role in learning in different fields. Researchers in a great number of studies have confirmed that students who have higher motivation are more successful in their learning [4–6]. Motivation is a major factor in the successful study of language acquisition [7] and can improve students’ academic performance [8]. Successful vocabulary learning is also important in learning and mastering a new language and there is an association between types of motivation including intrinsic motivation and students’ vocabulary knowledge [9].

Many researchers attempted to illuminate the motivational orientations of the participants of their studies. For example, female participants have been shown to be more intrinsically motivated [8]. Different scholars illustrated that participants were more instrumentally motivated [10, 11]. Knowing the orientation of learners can be illuminating since when educators know whether their students are more intrinsically motivated or extrinsically motivated or whether they follow more instrumental motives or integrative ones they can adjust their class activities and strategies to the benefit of their students. However, in the first place knowing the factors which influence the motivation of learners helps educators take their further steps in accordance with this precious knowledge. Even many studies have determined the effects of such factors as learning strategies, learner attitude, anxiety, academic performance, self-concept, self-determination, and learner background factors such as gender on intrinsic/extrinsic motivation [12, 13].

If an age group is shown to be more affected by intrinsic factors, educators can prepare learning materials that cater more to that preference. The study of the effects of field of study on intrinsic and extrinsic motivation can also clarify the potential barriers that prevent learners from reaching their potentials and help learners make more informed decisions about their academic paths. When students and instructors become informed about the effect of students’ work commitments on their intrinsic/extrinsic motivation, instructors will support their students in balancing work and academic issues more appropriately and students can follow personal development more effectively.

Though some studies address the impact of age [14, 15], there is still a paucity of research on the effects of such factors as the major and occupation on motivational orientation in all educational settings in general and in EFL contexts in particular. Studies that examined the effect of occupation on students’ motivation in educational settings are few and far between. The present study was an attempt to fill the gap and to eliminate the foregoing sparsity in an EFL context. If a study shows that the variable of major impacts the students’ intrinsic or extrinsic motivation, instructors are impelled to use diverse motivational strategies in classes with different majors. Moreover, if age has an influence over the motivation of students, in different classes with different age groups, again different strategies should be used for elevating students’ motivation. Conversely, if it is demonstrated that professional status has nothing to do with students’ intrinsic and extrinsic motivation, more similar motivational strategies can be used by educators. Thus, knowing about the impact that each learner background factor may exert on intrinsic vs. extrinsic motivation helps educators to set the stage for increasing the desired motivation in students of different fields of study, age and professional status and to follow the best strategies to boost the students’ intrinsic and extrinsic motivation. When educational practitioners are endowed with a knowledge of the factors that may influence their learners’ intrinsic/extrinsic motivation, they will be encouraged to follow more productive procedures. Accordingly, the results of the present study may go beyond previous reports, showing the influential variables which may exert impacts on the learners’ motivation.

The objectives of this study are threefold: to look into the impact of learners’ background factors of age, major (field of study), and professional status on their intrinsic and extrinsic motivation, to investigate the relationships between intrinsic, extrinsic and total motivation, and to determine the motivational themes. Since motivation is in many cases inextricably intertwined with language learning success, it can impact the quality and quantity of the rate of learning. Motivational factors can open inspiring horizons to learners. Knowing about the orientation of learners is illuminating, but awareness of the factors which are possibly influential in learners’ intrinsic or extrinsic motivation is a valuable piece of information which may cast new lights on further insightful aspects.

Recognizing these dynamics is far-reaching for a range of individuals involved in education, such as teachers, policymakers, and curriculum developers. For example, awareness of the differing motivational experiences across age groups can aid in creating age-appropriate learning resources and tailored educational strategies. Similarly, understanding the relationship between professional status and motivation can help educators follow techniques that cater to part-time students or adult learners balancing various obligations.

Although research focusing on the motivational orientations of learners offers valuable insights, it frequently falls short of linking these motivations to the factors which inform them. Understanding why students are intrinsically or extrinsically motivated can significantly impact how instruction is designed and delivered. For example, if a specific major is linked with heightened intrinsic motivation, educators within that discipline can foster more enriching learning experiences that resonate with students’ inherent interests. Acknowledging and addressing motivational factors not only improves the learning experience but also empowers students through connecting educational practices with their motivational needs and preferences.

Our study aims to address these identified gaps through a mixed-method approach. Moreover, this investigation raises three important questions that can guide instructional practices and help policymakers in developing initiatives and creating focused educational interventions. The following research questions are posed in this study:

1. How do learners’ background factors, such as age, major (field of study), and professional status, influence their intrinsic and extrinsic motivational orientation?
2. What are the relationships between intrinsic, extrinsic motivation, and total motivation indices?
3. Which motivational themes are revealed from interviews?

### Literature review

This section starts with definitions of motivation and is followed by some views on the impact of motivation on learning. Then, some previous studies which were conducted on motivation are elaborated and a theoretical framework is displayed.

### Motivation

#### Definition of motivation

Psychologically, motivation is an important component of human behavior and a multifaceted factor which up to now there has not been a consensus of opinions as to the meaning of this concept. Motivation provides the primary incentive to initiate learning the L2 and later the driving force to maintain the long and often tedious learning process [16]. It is the reasons and causes within a person. It is a kind of central mental engine which includes effort, cognition, and affect [17] and has some influences over an individual’s direction, intensity, and persistence of conscious act which [18].

Motivation is defined as an internal condition to arouse, direct, and maintain people’s goal-oriented approaches which plays a significant factor in students’ learning and success; while intrinsic motivation refers to engaging in activities for their inherent enjoyment, extrinsic motivation involves engaging in activities for external rewards [19]. The same definition is used in the present study.

#### Taxonomies of motivation

There are various taxonomies of motivation. A common categorization of motivation is integrative and instrumental. Integrative motivation is related to an intrinsic interest in the target language, the community and culture of its speakers together with an enthusiasm to acquire the language for the purpose of interacting and identifying with members of that community [19] and the purpose behind it is to know the culture and lifestyle of the target language for the goal of affinity with the target culture community. For example, an Asian family who immigrates to Canada wishes their child to know the target language to be able to communicate and get closer contact with people of the target country. Therefore, the family sends their child to an English-language school for integrative reasons. On the other hand, instrumental motivation helps language learners gain a benefit such as getting a better job or a higher salary.

#### Intrinsic/extrinsic motivation within the self-determination theory

Self-Determination Theory (SDT) connects personality, human motivation, and optimal functioning. It postulates that motivation is of two different types of intrinsic and extrinsic and both are strong forces in shaping who we are and how we behave [20] which come from some works on motivation [21] in the 1970s and 1980s. Intrinsic motivation refers to the internal factors and learning of language alone which helps the learners to achieve highly challenging tasks [22] without the need for incentives. It is the main key for persistence [23] and perseverance [24]. In contrast, extrinsic motivation is the purpose of getting something as an external factor like getting a prize. Intrinsic motivation roots from inherent and internal motives which come from within and are less related to the outside environment since intrinsically motivated learners are impelled to reach self-satisfaction. However, extrinsic motivation is connected with external factors and has its origin in one’s social status in relation to others and the surrounding environment; thus, extrinsically motivated individuals may strive to keep face and seek social prestige.

**Fig. 1:**
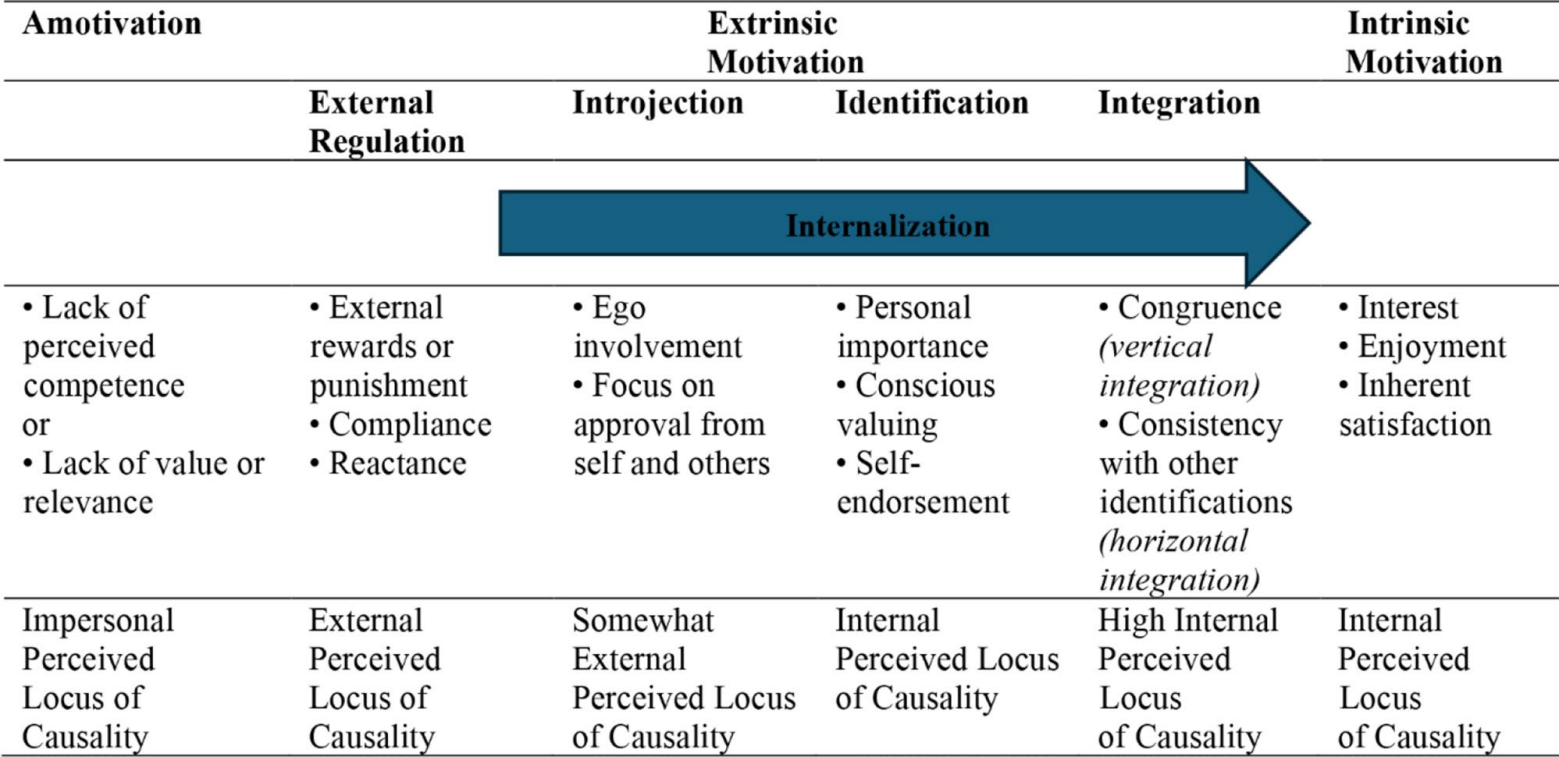
The Self-Determination theory showing types of motivation [25]

These two types of motivation can be considered within Self-Determination Theory. SDT research was initiated with a focus on intrinsic motivation. It is a prototypical expression [25] which presents the active integrative inclinations in human nature supposed by SDT [26]; this theory voices that the contrast is not straightforward due to the fact that instrumental motivations may differ extensively in content and character. SDT has four major subtypes of extrinsic motivation which are shown in Fig. 1.

External regulation has to do with behaviors which are guided by externally imposed rewards and punishments and is a form of motivation normally experienced as controlled and non-autonomous. However, interjected regulation is associated with extrinsic motivation which is in part internalized. In other words, behavior is controlled by the internal rewards of self-esteem for success and by prevention from anxiety, shame, or guilt due to failure. Identified and integrated motivations revolve around a sense of value, namely consider views about activities as worth the effort, even when they do not give them a sense of pleasure or enjoyment SDT [26].

The third subtype of extrinsic motivation is identification motivation. It includes paying attention to the behavioral aim to the extent that the action is approved or owned as personally important. Integrated regulation, as the most autonomous form of extrinsic motivation, happens when identified regulations are completely assimilated to the self. To put it another way, they have been assessed and made compatible with one’s values and requirements. Intrinsic motivation which is placed at the right of the continuum is the classic state which includes the doing of an activity for its innate contentment and is associated with interest, enjoyment, felt competence, and positive coping [27]. The following figure illustrates the Self-Determination Theory which incorporates different types of motivation.

Although intrinsic motivation has been connected with integrative motivation, extrinsic motivation is linked with instrumental motivation [28]. According to these definitions, the quality of an individual’s experience and performance can be different when one is acting for intrinsic versus extrinsic reasons. External expectations influence the individual’s extrinsic motivation and “the contrast between the extrinsic and intrinsic motivation is not a simple one, because instrumental motivations can vary widely in content and character” [26]; therefore, it is not easy to draw a dividing line. Self-Determination Theory deals with how learners are affected by their motivational needs and the learners will be more motivated when their needs are met. By studying the effects of the learner background variables of age, major and professional status, we get a clearer picture of how they influence university learners’ satisfaction of needs, as suggested by the Self-Determination Theory.

### Motivation and learning

#### Effects of motivation on learning

In the identity approach to language learning motivation, motivation is considered as a sociological construct which has a role in understanding how it can be connected with language learners’ situated experiences [29] in especial sociocultural environments. Motivation research has moved towards dynamic and contextual dimensions [30]. Therefore, by combining concepts of context, identity and complexity, motivation studies revealed how its main construct was a situationally dependent process and a state that is brief and temporary [3].

Boosted motivation could affect learning because enhanced motivation and attention could increase learning [31] as they promote the processes that underlie language learning. Motivation specifies what learners pay attention to when an interaction is taking place [32] and plays a prominent role in learning a new language. One of the language components which may be influenced by learners’ motivation is the vocabulary component. Within- and between-network approaches were applied using the Vocabulary Learning Motivation Questionnaire in measuring vocabulary learning motivation of Chinese learners [9]. The findings confirmed that different types of motivation were associated with students’ vocabulary proficiency. A positive correlation between motivation and using vocabulary learning strategies has also been reported [33].

It has been found that although instrumental and integrative motivation are related to the learners’ achievement, the former motivation is more correlated with the students’ achievement in learning English than the latter one [34]. Similarly, it is claimed that Iranian students have very high motivation and positive attitudes towards learning English, and they are more instrumentally motivated [11].

### Theoretical and conceptual foundations

#### Theoretical underpinning

This research is designed to investigate the impact of background factors, including age, academic discipline, and professional status on the intrinsic and extrinsic motivation of learners, by framing these inquiries through the lens of the Self-Determination Theory (SDT) and sociocultural theories of motivation. The conceptual framework, which is presented in the next section, outlines not only the theoretical elements of intrinsic and extrinsic motivation but also highlights how background variables interact with these motivational orientations. SDT addresses intrinsic and extrinsic motivation. The complexity of human motivation cannot be reduced to a simple dichotomy; it exists along a continuum [35]. According to SDT, fulfilling psychological needs increases intrinsic motivation, suggesting that learner background factors may play a vital role in how these needs are satisfied, thereby influencing their overall motivational landscape.

The conceptual framework of this research includes a synthesis of these theories, proposing a dynamic interplay between demographic factors and motivational orientations. It specifically posits that the age of learners may affect their motivation, a phenomenon attributable to shifting goals and values, as outlined by Carstensen (1992) in her Socioemotional Selectivity Theory [36]. Thus, as people age, they perceive the limitations of time, prompting them to care more about the way they spend their time, thereby motivating age differences in social preferences. This can affect their intrinsic and extrinsic motivation.

SST, which is a theory of life-span development, states that the approach of endings**–**whether due to aging or other purposes evokes motivational changes in which emotionally meaningful goals are preferred over exploration [35]. It suggests that age affects intrinsic and extrinsic motivation and as people age, their goals change from obtaining external rewards to prioritizing social and emotional needs which influences the levels of intrinsic and extrinsic motivation in multiple ways [37].

Socioemotional Selectivity Theory has implications for motivation in educational contexts based on learner background variables of age, major, and profession. Within the context of SST, intrinsic motivation is increasingly more important in older individuals. They are more inclined to engage in activities that involve meaningful engagement. Therefore, they often redirect their attention towards intrinsic motivation, placing a higher value on personal development and emotional gratification which is obtained from their learning experiences. Consequently, they are more prone to participate in learning for its intrinsic value rather than for external rewards. On the other hand, younger learners tend to set more future-oriented goals and are often stimulated by extrinsic motivation such as grades and career prospects. For older learners, the choice of major may align with their specific emotional goals and, in their selection, they are usually driven by intrinsic factors such as personal fulfilment. However, younger learners may follow external motives in choosing a major such as social prestige.

Likewise, older learners may be more intrinsically motivated in their profession, prioritizing jobs that give them emotional fulfilment and a sense of purpose over external rewards such as salary or status. In their professional journeys, they are attracted by positions that let them share their knowledge and expertise. This aligns with SST, which underlines that emotional and relational factors become more important with age. Conversely, students starting their careers often seek to establish themselves and pursue promotions and recognition which enhance their social status or bring them financial rewards.

Moreover, Self-Determination Theory asserts that when field of study is in line with a person’s values and goals, that exerts an effect on motivation. Thus, if a learner selects a major which they are interested in and is associated with their values and goals, they will probably experience intrinsic motivation; however, if students are put under pressure in the process of selecting their major, they may experience more extrinsic motivation [38].

#### Learner background factors and the conceptual framework

Reaching a conceptual model of factors can elucidate the effects of learner background factors on intrinsic/extrinsic motivation. Knowing what motivates people of different ages is of paramount importance [39, 40]. However, there are disparities as to the way experts measure different aspects of the construct of motivation. For example, it was held that extrinsic and intrinsic motivation should be measured separately [41]. Different scholars attempted to find the impacts of age on motivation. For example, elements of the employment relationship were examined [42] to see whether or not they varied significantly with age. They determined the relationship between age and motivation. Likewise, a bulk of data were collected on the satisfaction and importance of motivational factors [43] to specify if there were differences in their influences on older and younger knowledge workers.

Similarly, a multitude of experts conducted various investigations to clarify the potential effects of age on intrinsic motivation [14, 44, 45] and some experts performed studies to illuminate the out-turn of age on extrinsic motivational type [14, 39] and still others threw lights on the influences of age on intrinsic/extrinsic motivational orientation [46]. Others investigated the effects of major on motivation [47] or occupation on intrinsic motivation [48].

The impact of age was compared on Iranian EFL learners’ motivation level by comparing students whose ages ranged from 18 to 35 [49]. An inquiry was also conducted into the correlations between age and intrinsic/extrinsic work motivation [50]. Similarly, statistically significant relationships were found between work motivation and age [51]. The effect of different types of work-related motivations were also studied on age and work engagement [52].

Some other aspects such as field of study may exert some impact on motivation. Comparisons were made among the participants at different educational programs [47] giving considerations to their motivational complexions. Professional status may also affect the individuals’ motivation. Individuals working in different sectors were incorporated and their perceptions of the influence of intrinsic motivation were taken into consideration [48]. Teachers are the most important predictors of students’ learning motivation [53] and motivation in turn is a potential predictor of success. Motivation is one of the main factors that affect L2 learners’ success and performance in the language learning process [54] and EFL learners can increase their productivity by knowing the way they learn because “by being aware of how you learn, you can become a more productive and impressive student” [55] p72. Moreover, EFL teachers can obtain positive outcomes by adjusting their teaching ways to the needs of their students [56]. The impact of Japanese individuals’ intrinsic and extrinsic motivation has been studied on work engagement [57] and the relationship between intrinsic and extrinsic motivation and engagement have also been explored which showed that the two types of motivation are related to work engagement as there was a positive relationship between both intrinsic and extrinsic motivations and work engagement [58].

**Fig. 2:**
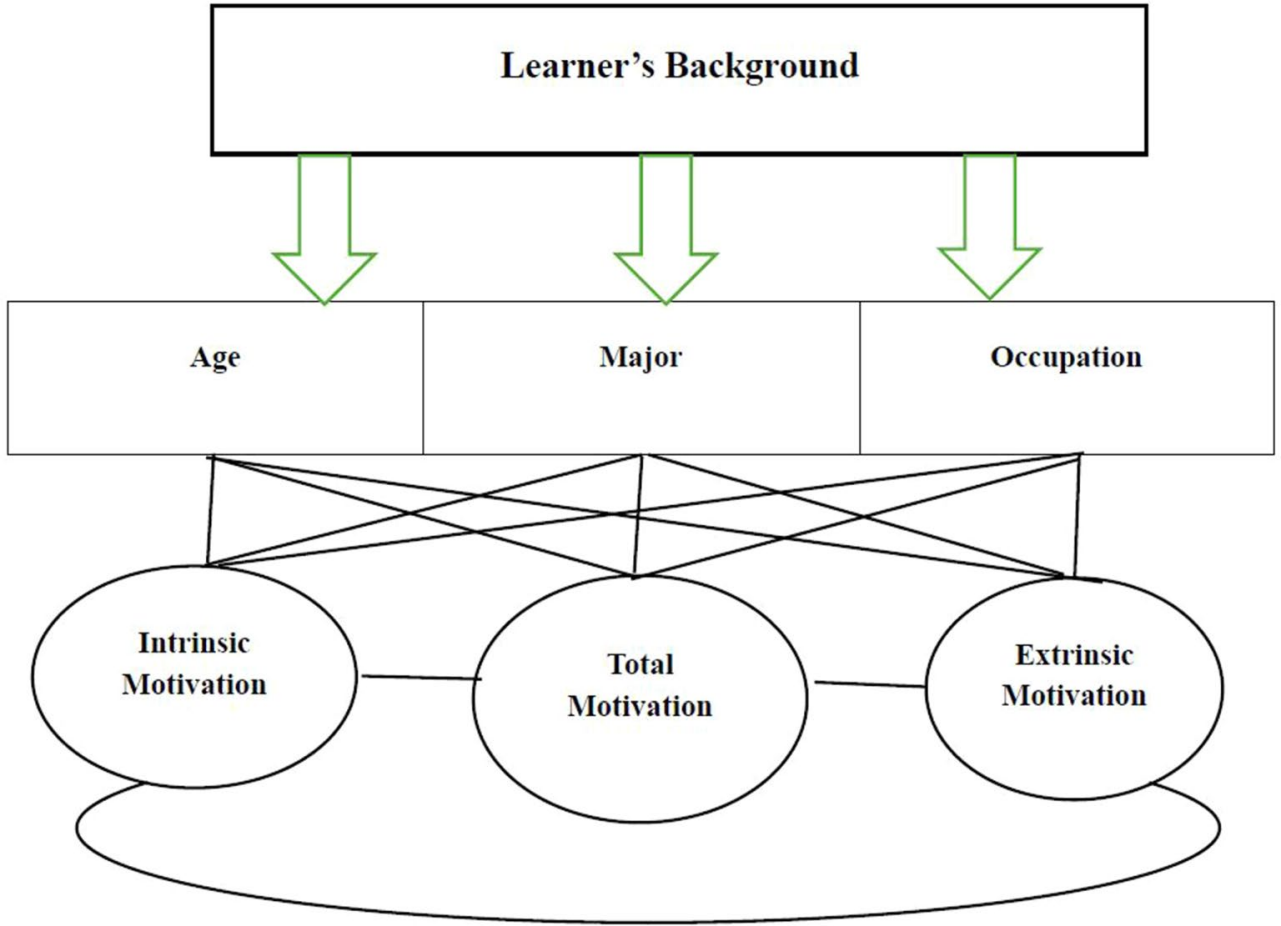
Primary conceptual model of factors affecting intrinsic/extrinsic motivation

**Fig. 3:**
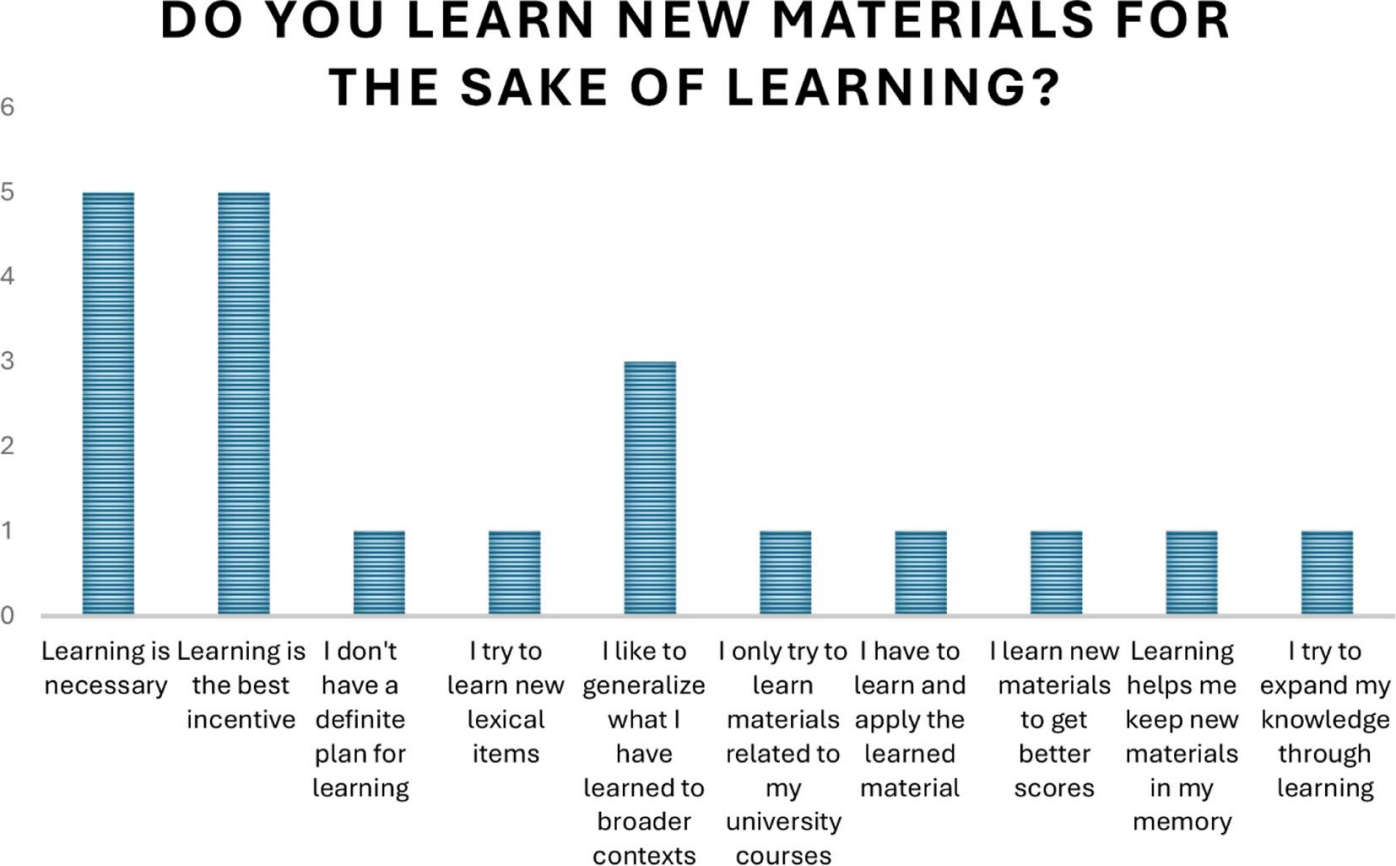
Students’ interview response

This study is intended to reach a conceptual model of factors influencing intrinsic vs. extrinsic motivation in an EFL context. There are many factors which may affect the foregoing types of motivation. The variables in this study which were more particularly related to motivation were age, major, and occupation. They were involved in the present study because they are believed to have an impact on EFL learners’ intrinsic vs. extrinsic motivation. The primary conceptual framework of the study is presented in Fig. 2. The lines drawn from the variables of age, major, and occupation towards the variables of intrinsic, extrinsic, and total motivations show the possible effects of the background variables on motivational types. The final conceptual framework is illustrated in Fig. 3 at the end of the Discussion section.

The conceptual model of factors in this study are considered within an EFL context. Although English as a Second Language (ESL) and English as a Foreign Language (EFL) have some common features and the two terms are usually used interchangeably, there are some evident differences between the two approaches. English as a Second Language, or ESL, is a term typically used in Canada, Australia, and the United States to talk about people who come to the country with another primary language and learn to speak English secondarily; while it is also sometimes used to speak of people who live in a country where English is the official language, but most of the population speak another native tongue. However, English as a Foreign Language, or EFL is related to learning English in countries such as Iran, Indonesia and China where English is not the spoken language. In ESL settings, learners have more opportunities to communicate with L2 speakers which is an important element in second language learning in comparison with EFL learners [59]. While in EFL settings, motivation is usually more extrinsic and students do not have real-life access to English, in ESL contexts, intrinsic motivation is stronger as English is more relevant to students’ daily lives [60]. However, some studies maintained that this is not always the case as in a descriptive-diagnostic study on how EFL students become motivated, it was shown that EFL students had high levels of intrinsic motivation [61].

When educational practitioners know about the significant variables in enhancing students’ motivation, that forms their core competency. When an educator knows the way age groups influence learners’ intrinsic or extrinsic motivation, he/she may include more suitable activities for various classes of different ages. That also stands up for learners of different major and profession since understanding the way they impact students’ intrinsic or extrinsic motivation helps instructors vary class activities accordingly. The grasp of this invaluable knowledge is accelerated and facilitated through the implementation of the current study.

## Methodology

In the methodology section, the research design which is used in this study is specified. Then, the participants who were involved in the study are discussed followed by data collection procedure, research instruments and data analysis procedure.

### Research design

This study employed an explanatory sequential mixed-methods approach to enhance the depth and breadth of research findings. The quantitative research focused on discovering relationships among learner background variables such as age, major, and professional status. Complementing this, qualitative findings aimed to provide a richer understanding by elucidating the motivational trends which were identified through interviews.

While both research types have inherent merits and limitations, the chosen solution is a mixed-methods design integrating the advantages of both qualitative and quantitative methods for a more comprehensive research outcome. An explanatory sequential mixed-methods design entails two distinct phases: a quantitative phase followed by a qualitative phase [62]. Thus, in our study, we first gathered and analyzed our quantitative data which was followed by the collection and analysis of qualitative data. This sequential process allows for a nuanced exploration of the research questions, ensuring a thorough and cohesive examination of the learners’ background factors and their influence on intrinsic and extrinsic motivational orientations. In this mixed method study, participants consisted of students of Shiraz Farhangian University and the research instruments used were a motivation questionnaire and interviews. The qualitative data which handle the role of university students’ motivation and specify whether or not selected students are more driven by intrinsic motives or extrinsic ones will complement our quantitative findings.

### Participants

The participants comprised university students from Shahid Bahonar Farhangian University of Shiraz, Iran. These students were having their English courses. The researchers utilized cluster sampling method. It is a sampling procedure in which the entire population of interest is divided into groups, or clusters which are of predeterµined nuµbers and they randomly selected and sampled from the clusters and collected data from each individual unit in the selected clusters [63].

As all the units within a cluster were selected, the sampling procedure in this study is one-stage cluster sampling. All students studying at Shahid Bahonar Farhangian University are female students. Therefore, only female students were involved in the present study. In accordance with our sampling procedure, each class is deemed as a cluster. Data were collected from every unit in sampled clusters. Students of 4 classes were randomly selected from classes of different fields of study. Based on a standard table offered by Krejcie and Morgan, we had to involve around 97 participants as 130 students comprised the total population out of which we selected the sample (See Krejcie and Morgan’s Sample Determination Table in the appendix). As we predicted a few dropouts, to increase the reliability of our findings, we decided to include 100 Bachelor’s degree students at the initial stage. Throughout the study, a few participants withdrew, leaving us with a final group of 94 students who completed the research and remained as participants till the end of the study. Consequently, the sampling approach not only provided a representative sample of the population but also followed a methodical selection process that focused on incorporating from different academic disciplines. The first cluster included 37 students whose major was Elementary Education and who were students of the same class, but one of students dropped out of the study. The next cluster (class) incorporated 20 students who were studying Educational Affair. The third and fourth classes included 20 and 23 students studying Social Sciences and Theology and Arabic, respectively. Six students dropped out of the study in later stages. Those who remained in the study were students of four classes from the different majors of Elementary Education (*N* = 36), Educational Affairs (*N* = 19), Social Studies (*N* = 17), and Theology and the Arabic language (*N* = 22). The percentage of participants is illustrated in Fig. A.1 in the appendix.

The students were of four different age groups: four participants were 29 years or below, 36 were aged between 30 and 39, 48 were in the age range of 40–49, and 6 were 50 years or above. The percentage of participants of different age is demonstrated in Figure A.2 in the Appendix. All of the participants were both working and studying. The participants represented various occupations: 54 were elementary school teachers, 32 were secondary level school teachers, and 8 were assistants for educational affairs.

### Tools

The tools used in this research were Harter ‘s (1981) questionnaire [64] and a semi-structured interview incorporating questions related to the students’ motivation. Harter’s intrinsic versus extrinsic orientation scale has proved an extremely important addition to the field [65], largely because of the noticeable developmental trends it has shown. The questionnaire has some subscales to represent intrinsic vs. extrinsic motivation. Each subscale contains items which measure an individual’s motives for engaging in different activities. The selection of Harter’s (1981) questionnaire for our investigation into intrinsic and extrinsic motivation among university students was a deliberate choice based on various important factors and the rational for our choice are explicated in the following paragraphs.

In our study, we considered intrinsic/extrinsic motivation within self-determination theory, and Harter’s instrument is rooted in that theory which is a well-accepted framework in motivation research. SDT illuminates the fact that intrinsic and extrinsic motivation are distinct constructs which are affected by a series of factors, including individual learner traits. We also planned to investigate intrinsic and extrinsic motivations as separate concepts in our study and reviewed several other scales as well. However, Harter’s inventory was the optimal one for the purposes which we followed. The questionnaire delineates between intrinsic and extrinsic motivation and includes a thorough exploration of these two aspects of motivation; this distinction is crucial for our research objectives, enabling a complete understanding of how these motivational types coexist and interact with the learner background factors of age, major, and profession.

According to SDT, motivation increases when individuals’ needs for autonomy, competence, and relatedness are met [26], and Harter’s questionnaire can clarify how intrinsic/extrinsic motivations are related to the fulfilment of these needs, providing support for SDT. In other words, as SDT is widely applied in educational research, the questionnaire used in our study helps to bridge the gap between theoretical frameworks and practical applications.

Additionally, we aimed to explore the intricacies of motivation; thus, the use of a tool, which is connected with established theories, can guarantee the relevance and rigor of our study. This questionnaire is adjustable to different learning contexts and its broad range of items facilitates in-depth data analysis; it is constructed in such a way that makes it appropriate to be used in various settings including educational institutions, workplaces, industries, etc. Thus, it has practical application and is not of limited use.

This questionnaire is a standardized instrument for measuring individual differences in motivation which has been widely used and validated in different contexts and populations, making it a dependable instrument for assessing intrinsic and extrinsic motivation. The long use of the scale allows researchers to make comparisons and contrasts with earlier studies, thereby identifying changes or consistencies in motivational trends through time. For our research, the confirmed reliability with a Coefficient alpha of 0.74 adds to the credibility of our quantitative finding. On that account, it has been used as a potentially suitable instrument in our study and has been double-checked in the EFL context in which the study was conducted which will be explained in the next section. Moreover, the questionnaire features a straightforward five-point Likert scale that simplifies the responses provided by participants. Participants in our study appreciated the questionnaire’s simplicity, enabling them to offer more reflective responses. The aforementioned merits of the inventory are pertinent to the objectives of our study and enhance our understanding of significant issues in learning environments.

The questionnaire involved 33 items scored using five-point Likert scale format ranging from ‘never’ to ‘always’. We used this questionnaire as we needed a standard self-report scale to tap the selected students’ intrinsic versus extrinsic orientation and to examine how intrinsic vs. extrinsic motivation might be influenced by learner background factors including major, age, and occupation. Out of the 33 questionnaire items, the first 17 items were related to intrinsic motivation, while the last 16 items dealt with extrinsic motivation. For measuring the indices, the scores for intrinsic and extrinsic motivation statements were added up and an average was estimated by dividing the total scores by the number of statements in each category. To calculate the total motivation index, all the items were taken into consideration and the sum was divided by the total number of statements to give a numerical representation of motivation.

Semi-structured interviews were held and interview guides were applied. A set of questions were prepared as interview guides which provided organization to the interview by knowing what to ask and in what order. Similarly, it structured our interviews in a way that they were easy to follow and moved smoothly from one question to another to help the conversation flow and to reach flexibility and maximum information gathering. Thus, our questions were listed together with some follow-on prompts to gather more detailed information. The selected students were assured that their responses were only for research purposes and would remain completely confidential.

### Reliability and validity of instruments

The reliability of the questionnaire was calculated through Cronbach’s alpha which is a measure of internal consistency. The alpha index for the whole questionnaire was 0.74. Even though the questionnaire has previously been checked for validity in both EFL and ESL contexts in multiple ways, it was validated again in this study by several professors and experts in the field. It was given to a number of experts and the validity, accuracy and fluency of the questionnaire were checked. Moreover, the categorizations of themes related to interview data were given to three experts to specify their agreement or disagreement with the categorization of each theme. Member checking was also used to establish the rigor of the interview data. The data obtained from interviews were transcribed and sent back to the interviewees for member checking. Interviewees read the transcriptions and verified their content.

In order to estimate the interrater reliability of the interviews, Cohen’s kappa was used. Cohen’s kappa coefficient is a statistical measure of inter-rater agreement (reliability) used in qualitative research. The formula used for the degree of agreement was as follows:

$$\:{K}=\frac{{f}{a}-{f}{c}}{{N}-{f}{c}}$$

OR

$$\:K=\frac{Number\:of\:agreements-Half\:of\:total\:number\:of\:themes}{Total\:number\:of\:themes-Half\:of\:total\:number\:of\:themes}$$

Cohen’s Kappa values were calculated for each of the raters. Zero signifies lack of agreement while 1 designates total agreement among raters. The average Kappa value for interviews was 0.79. The value reflects a rather high strength of agreement and reliability of interview data. The results related to interviews are illustrated in the following Table 1.

**Table 1:** Cohen’s Kappa values for themes revealed by interviews

	Expert 1	Expert 2	Expert 3	Average Kappa Value
Agreed Kappa Value	0.78	0.84	0.75	0.79

### Procedure

All the participants were selected from one level of education referred to here as undergraduate (B.A. students). A few days before the days of administration, the researchers of the study talked about the survey and the interviews with the instructors of the classes. These classes were the ones which were randomly selected by cluster sampling. The instructors were assured that these procedures were related to an approved research project. The instructors were all helpful and the researchers distributed the paper questionnaires inside the classes. When the questionnaire was administered to students, they were asked to answer the questions as carefully as possible in twenty minutes.

One fourth of the university students who participated in the study and received the research questionnaire were randomly selected to attend semi-structured interview sessions. In each instructor’s class list, in addition to students’ names, numbers attributed to students are also shown. The researchers who acted as the interviewers took the lists in the selected classes (with instructors’ permission) and chose some names on the lists by drawing some random numbers. The randomly selected interviewees were to be included in the interviews upon their approval. Interviewees were from the four different majors of Elementary Education, Educational Affairs, Social Studies, and Theology and the Arabic language. The interviews were carried out individually in Persian, and students’ voices were recorded and later transcribed by the researchers. Inside their classes, the interviewees answered the interview questions when their university classes ended. The interview time was arranged a few days before the interviews. The interviews were recorded to save all the responses given by the interviewees.

### Ethical considerations

All study participants were informed about the nature of our research. They were provided with sufficient information about research purposes, and they were provided with the opportunity to pose the questions that came to their minds. They were also assured that all the information which they supplied would be confidential and complete anonymity would be maintained since their identities would remain unlinked to the data in study reports.

Participation in the research was voluntary, enabling the participants to withdraw at any point without facing negative consequences. While carrying out the semi-structured interviews, the researchers felt obliged to help interviewees feel comfortable talking about their motivations and personal backgrounds. No pressure was exerted on interviewees to disclose personal information which they were reluctant to share. The researchers also adhered to ethical guidelines regarding the storage and management of data throughout the research process. Data were collected and securely stored, with access restricted to authorized personnel.

### Data analysis

Descriptive statistics of frequencies, means, and standard deviations (SDs) were used to summarize data and highlight potential relationships between the variables in our study. Quantitative data were analyzed using MANOVA to determine the effects of different variables of major, age and occupation on students’ motivation. Since MANOVAs offers the advantage of simultaneous assessment of multiple continuous dependent variables and our study included the dependent variables of intrinsic motivation, extrinsic motivation and total motivation, it was the most suitable statistical technique for our study. Moreover, to check the degree of linear dependence between the variables, we used Pearson correlation test. Qualitative data were analyzed using the SPSS version 23 and Excel software, and different coding procedures were applied on them.

To reduce the probability of Type I error, we used multiple analysis of variance (MANOVA). In this study, Wilk’s Lambda was used which showed the quantity of variance accounted for in the dependent variable by the independent variable. In addition to MANOVA, Pearson Product moment correlation was used to demonstrate the linear relationships that existed between the sets of data. For running this type of correlation, there is an assumption that variables are on either interval or ratio scale and they are continuous.

The empirical material collected underwent a comprehensive thematic analysis, employing Attride-Stirling’s (2001) thematic networks technique [66] which guided the extraction of lowest-order premises, termed Basic Themes, from the text. These Basic Themes were then organized into classes, forming more abstract principles known as Organizing Themes. Furthermore, super-ordinate Global Themes were identified, encapsulating the primary metaphors embedded in the text.

To execute this thematic analysis, we initiated Attride-Stirling’s meticulous coding process for categorizing the interview data, developing a systematic coding framework. Subsequently, we dissected the text segments using this framework, identifying abstract themes that were then refined for clarity and precision. Thematic networks were constructed by strategically arranging and selecting basic themes which represented specific ideas. They were the first layer of the coding framework and included the lowest-order premises from our interview data. Thus, meaningful segments of data were labeled; the codes contained the essence of the content. These basic themes were synthesized and grouped into broader higher-level categories termed organizing themes. Therefore, the underlying patterns and abstract principles that encapsulated the responses of the interviewees were created by merging related themes into broader categories while looking for patterns among them. The deduction of global themes added a layer of depth, representing the main metaphors within the text that captured the essence of participants’ motivations and experiences. These overarching themes which summarized the findings offered a holistic perspective on the qualitative data, representing the principal motivational currents present in the interviews. Visualized as intricate web-like representations, these thematic networks were meticulously confirmed and refined, culminating in a cohesive and systematic representation of the qualitative data analysis.

## Results

The first section is dedicated to results of quantitative data analysis through MANOVA and statistical procedures of Pearson Product Moment correlation and the second section deals with the results which are related to qualitative data analysis conducted via thematic coding-procedures.

### Quantitative results

In the following sections, the results connected with the first two research questions are provided. To address the research questions under the categories of the dependent variables and independent variables (major, age and occupation), we analyzed the data using Multiple Analysis of Variance (MANOVA).

#### Research question 1 how do learners’ background factors, such as age, major (field of study), and professional status, influence their intrinsic and extrinsic motivational orientation?

##### Major

Data obtained from Iranian students of different majors revealed that the error variances of the dependent variables were equal across the groups, and the assumptions of homogeneity of error variances were not violated among the groups. The results in Table 2 show the findings of the Levene’s test of equality of variance.

**Table 2:** Levene’s test of equality of error variance

F	df1	df2	Sig.
2.008	3	90	0.118
0.671	3	90	0.572
0.554	3	90	0.647

As there may be an interplay between intrinsic and extrinsic motivations, in this study not only the effects of learner background factors are estimated on intrinsic and extrinsic motivations but the impacts of the total motivation index are also determined by summing the scores of the individual items across different subscales (dimensions) of motivation. The whole motivation score shows the total level of motivation across various aspects of intrinsic and extrinsic motivations. To analyze the differences between the students of different majors, we estimated the means. The means and standard deviations of different groups are presented in Table 3. Students majoring in Theology and the Arabic language obtained the highest means in intrinsic motivation and total motivation index; however, students of Social Studies showed the highest means in extrinsic motivation. To check whether the differences were statistically significant, we conducted multivariate test on the variable major.

**Table 3:** Means and standard deviations of students of different majors

	Major	Mean	Std. Deviation
Mmot	Elementary Education	3.3190	0.28816
	Educational Affair	3.3349	0.45572
	Social Studies	3.4314	0.49429
	Theology and Arabic	3.4490	0.52816
	Total	3.3730	0.42413
Mintrinsic	Elementary Education	3.7794	0.52939
	Educational Affair	3.6997	0.54898
	Social Studies	4.0242	0.64775
	Theology and Arabic	3.8877	0.89926
	Total	3.8329	0.65630
Mextrinsic	Elementary Education	2.8299	0.62760
	Educational Affair	2.9474	0.75094
	Social Studies	2.8015	0.73926
	Theology and Arabic	2.9830	0.62802
	Total	2.8843	0.66782

The results of the multivariate test are shown in Table 4. The test yielded a Wilks’ Lambda = 0.725, F (6, 178) = 0.607, and *p* = .725. It seems that EFL students’ major did not have any effects on intrinsic, extrinsic, and total motivation indices

**Table 4:** Multivariate test of motivation by major

Effect	Value	F	Hypothesis df	Error df	Sig.	Partial Eta Squared	Observed Power
Pillai’s Trace	0.040	0.613	6.000	180.000	0.720	0.020	0.241
Wilks’ Lambda	0.960	.607^b^	6.000	178.000	0.725	0.020	0.238
Hotelling’s Trace Roy’s Largest Root	0.041 0.29	0.601 .870^c^	6.000 3.000	176.000 90.000	0.729 0.460	0.020 0.028	0.236 0.233

##### Age

Table 5 demonstrates that Levene’s test of homogeneity of variance was not statistically significant for the dependent variables and shows the findings of Levene’s test which was used to check for the homogeneity of co-variance matrices of the dependent variables of the study. Since the error variances of the dependent variables were equal across the groups, and the assumptions of homogeneity of error variances were not violated among the groups, MANOVA was used to analyze the level of differences among university students of different age groups.

**Table 5:** Levene’s test of equality of error variances

	F	df1	df2	Sig.
mmot	2.008	3	90	0.118
mintrinsic	0.671	3	90	0.572
mextrinsic	0.554	3	90	0.647

To analyze the differences among the students of different age groups, we used descriptive statistics. Table 6 presents the means and standard deviations of different groups. As shown, EFL students in the age range of 50 and above obtained the highest vector of means. The students who were aged 40 to 49 years obtained the lowest vector of means when compared with those of other age groups. A MANOVA mixed-group design (group × measures) was conducted to determine the effect of independent variables on the dependent variables.

**Table 6:** Means and standard deviations of EFL students of different age groups

	Age	Mean	Std. Deviation	*N*
mmot	50 and Above	3.5556	0.56029	6
	40 to 49	3.3289	0.34290	48
	30 to 39	3.4049	0.50883	36
	29 and Below	3.3409	0.28143	4
	Total	3.3730	0.42413	94
mintrinsic	50 and Above	4.1078	0.59341	6
	40 to 49	3.7953	0.55048	48
	30 to 39	3.8219	0.80881	36
	29 and Below	3.9706	0.43093	4
	Total	3.8329	0.65630	94
mextrinsic	50 and Above	2.9688	1.05531	6
	40 to 49	2.8333	0.62717	48
	30 to 39	2.9618	0.67319	36
	29 and Below	2.6719	0.57594	4
	Total	2.8843	0.66782	94

A MANOVA mixed-group design (group × measures) was performed to determine any effect of age on the dependent variables. The multivariate test was performed on the data at the 0.05 level of significance. Findings from the multivariate test of Wilk’s Lambda showed that the main effect of age was not significant (Wilks’ Λ = 0.970, F (6, 178) = 0.457, *p* = .839). It means that age did not influence the selected participants’ intrinsic, extrinsic, and total index of motivation. The detailed results are shown in Table 7.

**Table 7:** Multivariate test conducted for motivation by age

Effect		Value	F	Hypothesis df	Error df	Sig.	Partial Eta Squared	Partial Eta Squared
Age	Pillai’s Trace	0.030	0.462	6.000	180.000	0.836	0.015	
	Wilks’ Lambda	0.970	.457^b^	6.000	178.000	0.839	0.015	1.16
	Hotelling’s Trace	0.031	0.452	6.000	176.000	0.843	0.015	

**Professional status**.

Another factor which could influence the students’ motivation was their occupation. This factor was classified into elementary school teacher, secondary school teacher, and assistant for educational affairs. Data obtained from the students of different occupations revealed that the assumptions of homogeneity of error variances were not violated. Table 8 shows the results of Levene’s test of equality of variance for EFL students of different occupational status.

**Table 8:** Levene’s test of equality of error variances

	F	df1	df2	Sig.
mintrinsic	0.955	2	91	0.389
mextrinsic	0.589	2	91	0.557
mmot	2.400	2	91	0.096

To analyze the differences between the students of different occupations, we computed their means. Table 9 illustrates the descriptive statistics of means and standard deviations for Iranian students. Secondary school teachers obtained the highest means in intrinsic motivation and total motivation index. Nevertheless, assistants of educational affairs showed the highest means in extrinsic motivation.

**Table 9:** The means and standard deviations of students with different occupations

	Occupation	Mean	Std. Deviation	*N*
mintrinsic	Elementary School Teacher	3.7462	0.51609	54
	Secondary School Teacher	4.0551	0.81123	32
	Assistant for Educational Affairs	3.5294	0.63433	8
	Total	3.8329	0.65630	94
mextrinsic	Elementary School Teacher	2.8356	0.63486	54
	Secondary School Teacher	2.8770	0.69570	32
	Assistant for Educational Affairs	3.2422	0.75181	8
	Total	2.8843	0.66782	94
mmot	Elementary School Teacher	3.3047	0.32400	54
	Secondary School Teacher	3.4839	0.51059	32
	Assistant for Educational Affairs	3.3902	0.58992	8
	Total	3.3730	0.42413	94

MANOVA was conducted to determine the effect of Iranian students’ occupation on intrinsic, extrinsic, and total motivation index. Findings yielded a non-significant Wilks’ Λ = 0.909, F (4, 180) = 2.2, and *p* = .071 for the dependent variables. In other words, professional status did not impact the participants’ intrinsic, extrinsic, and total index of motivation. The results of the multivariate test are shown in Table 10.

**Table 10:** Multivariate test for intrinsic motivation, extrinsic motivation, and total motivation index by occupation

Effect		Value	F	Hypothesis Df	Error df	Sig.	Partial Eta Squared	Observed Power
Occupation	Pillai’s Trace	0.093	2.211	4.000	182.000	0.069	0.046	0.642
	Wilks’ Lambda	0.909	2.200^b^	4.000	180.000	0.071	0.047	0.639
	Hotelling’s Trace	0.098	2.188	4.000	178.000	0.072	0.047	0.636
	Roy’s Largest Root	0.074	3.348^c^	2.000	91.000	0.040	0.069	0.619

#### Research question 2. What are the relationships between intrinsic, extrinsic motivation, and total motivation indices?

**The Interrelationships between motivation types**.

Pearson Product Moment Correlation was also used. Since one of the pre-conditions of this type of correlation is that variables are on either an interval or a ratio scale, the variables of intrinsic motivation, extrinsic motivation, and total motivation index were taken into consideration. Table 11 shows the results of correlation.

**Table 11:** Correlations

		mextrinsic	mintrinsic	mmot
mextrinsic	Pearson Correlation	1	− 0.179	0.620^**^
	Sig. (2-tailed)		0.084	0.000
	N	94	94	94
mintrinsic	Pearson Correlation	− 0.179	1	0.660^**^
	Sig. (2-tailed)	0.084		0.000
	N	94	94	94
mmot	Pearson Correlation	0.620^**^	0.660^**^	1
	Sig. (2-tailed)	0.000	0.000	
	N	94	94	94

**. Correlation is significant at the 0.01 level (2-tailed)

The Pearson product-moment correlation showed that there was a significant and positive relationship between intrinsic motivation and total motivation index (*r* = .660, *p* < .01). Moreover, there was another significant, positive, and strong correlation between extrinsic motivation and total motivation index (*r* = .620, *p* < .01). On the contrary, there was a negative correlation between extrinsic and intrinsic motivation (*r* = − .361); however, it was not statistically significant.

### Qualitative results

#### ***Research question 3. Which motivational themes are revealed from interviews?***

A complete thematic network was developed while analyzing the data which were collected from the interviews with selected Farhangian University students. The collected empirical material was analyzed using a thematic analysis, aided by a thematic network called Attride-Stirling’s thematic networks technique. These networks organize the extraction of lowest-order premises discernable in the text (Basic Themes), classes of basic themes grouped together to array more abstract principles (Organizing Theme) and super-ordinate themes covering the main metaphors in the text (Global Themes). The results of the foregoing thematic network and the selected students’ interview responses are provided in Table 12; Fig. 3, respectively.

To do the thematic analysis using Attride-Stirling’s thematic networks analysis, we first coded the interview data by devising a coding framework and dissecting the text segments using that framework. In the next step, abstract themes were identified from coded text segment and the themes were refined. Then, thematic networks were developed by arranging themes, choosing basic themes, rearranging into organizing themes, deducing global themes, illustrating themes as web-like representations and confirming and refining the networks. In the next steps, thematic networks were explored and later summarized and interpreted.

Table 12 presents a thematic network that categorizes the responses of interviewees concerning their motivations to learn new materials, as elicited during semi-structured interviews. The responses are organized into three main levels of themes of basic themes, organizing themes, and global themes. Basic themes are specific phrases directly taken from our interview data. They highlight the different reasons respondents provided for their motivation to learn.

For example, the basic themes of “for more satisfaction” and “because I am interested” which are the lower-order premises are directly obtained from interview data. These two themes are merged into the broader theme of “interest/enjoyment” as the organizing theme which is a higher-order category. Then, the super-ordinate theme of “internal rewards as intrinsic motivation factors” is created to summarize the findings and synthesize the insights obtained from both basic and organizing themes. In this study, we extracted the two global themes of “internal rewards as intrinsic motivation factors” which was related to intrinsic motivation factors and reflected the essence of interviewees’ motivations that originated from internal satisfaction and “external rewards as extrinsic motivation factors” which incorporated various motivations fueled by external approval and tangible rewards.

**Table 12:** Thematic network

Basic Themes	Organizing Themes	Global Themes
For more satisfaction	Interest/Enjoyment	Internal rewards as Intrinsic Motivation Factors
Because I am interested		
For more effectiveness	Value/Usefulness	
Because I want to learn more lexical items	Learning	
I want to expand my knowledge		
I plan to review my lessons		
I like to apply different learning methods		
I don’t plan to be praised by others	Perceived Choice	
Because I want to teach others	Knowledge Transfer	
For effective communication	Relatedness	
For a better life	Effect/Importance	
For more success		
For better grades	Scores	External rewards as Extrinsic Motivation Factors
I like to be praised by others	Praise	
Learning is necessary	Value/Usefulness	Internal rewards as Intrinsic Motivation Factors
Learning helps me keep new materials in my memory		
Learning is the best incentive	Effect/Importance	
I don’t have a definite plan for learning	Ego Involvement	
I try to learn new lexical items	Learning	
I like to generalize what I learned to broader contexts	mastery	
I have to learn and apply the learned material		
I try to expand my knowledge through learning		
I learn new materials to get better scores	Scores	External rewards as Extrinsic Motivation Factors
Motivation is of paramount importance	Effect/Importance	Internal rewards as Intrinsic Motivation Factors
Motivation enhances learning		
Motivation facilitates learning		
Motivation leads to improvement in learning		
Motivation cannot be applied in real life	Relatedness	
It also depends on teaching methodology		
Motivation increases enthusiasm	Value/Usefulness	
Learning English spelling can also be of great help		
Professors can help to increase learning	Learning	
Motivation can improve my social status	Improved Social Status	External rewards as Extrinsic Motivation Factors

**Fig. 4:**
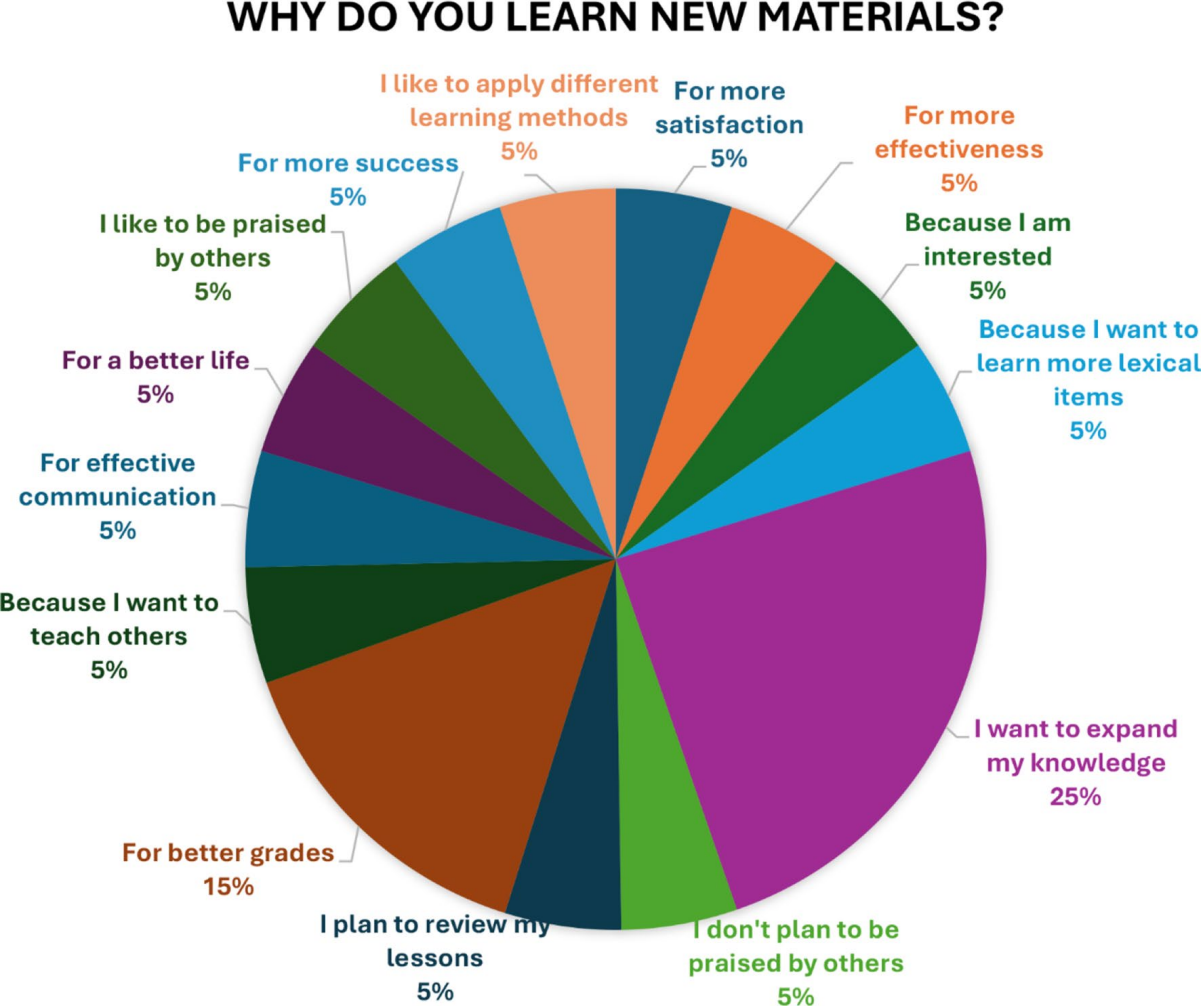
Students’ interview responses

Qualitative data were transcribed and refined through some coding procedures. Using Excel and SPSS version 23, we analyzed the results and some themes emerged from the interviewees’ responses. One of the main interview questions was “why do you learn new materials?” The responses showed university students’ motivational orientations. Though they provided various explanations, the most common response obtained indicated that they were eager to expand their knowledge (Table A.1 in the appendix and Fig. 4).

Another main interview question sought to find out the reasons why interviewees learn. Again their responses were put in the form of transcriptions, using Excel and SPSS. It was demonstrated that the most frequent themes were that the interviewees learned new materials because they thought learning was necessary and it was the best motivation. (See Table A.2 and the Figure A.1 in the appendix).

**Fig. 5:**
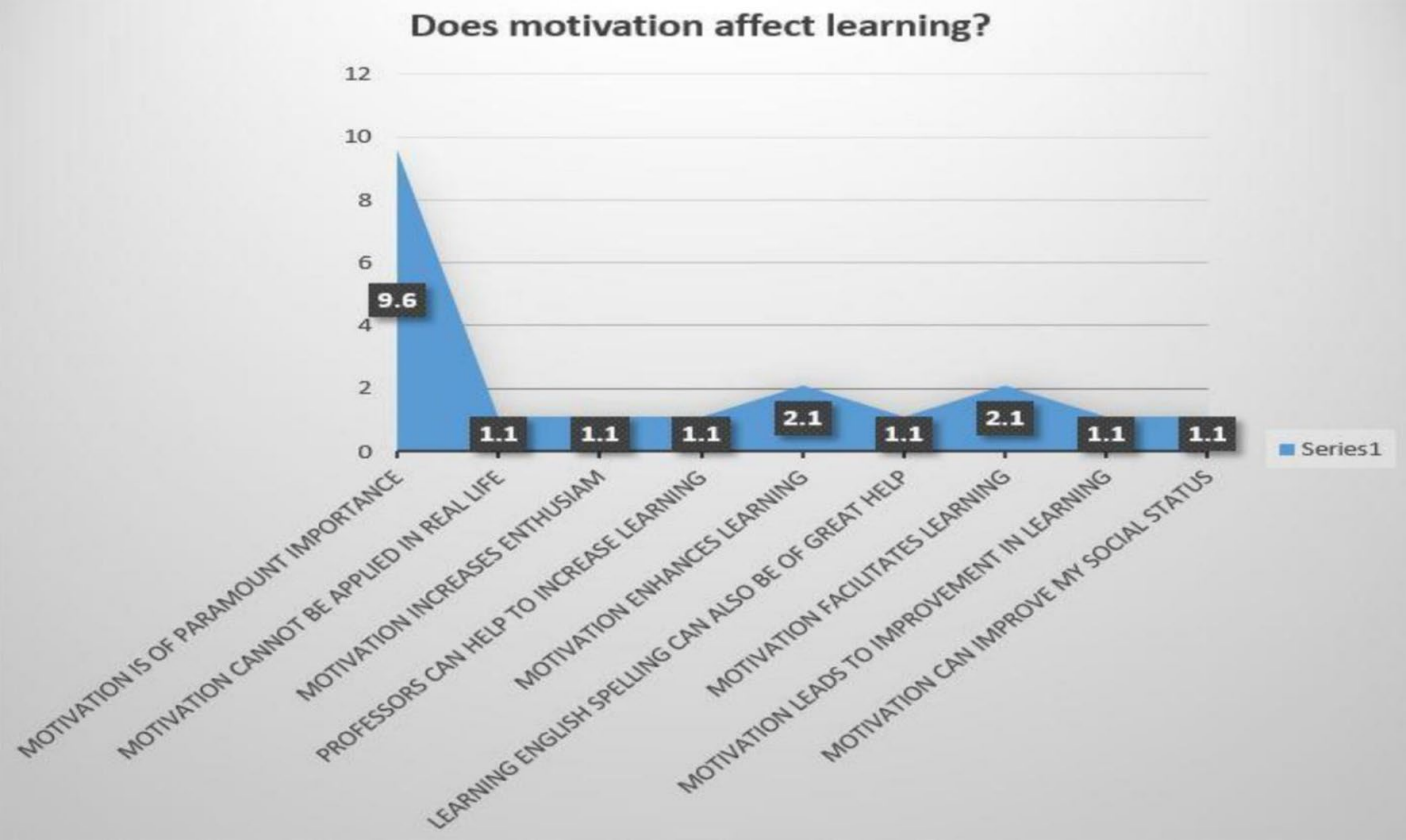
Students’ interview response

The twenty interviewees were asked another main interview question which intended to clarify the interviewees’ beliefs regarding the role of motivation in learning. Various responses were obtained and different themes emerged. The analysis of the results revealed that the most frequent responses showed that the interviewees considered motivation as something of great importance and that they thought that motivation increased learning and made learning much easier. (See the following Table 13; Fig. 5).

**Table 13:** Students’ responses to “does motivation affect learning?”

		Frequency	Percent	Valid Percent	Cumulative Percent
Valid	Motivation is of paramount importance	9	9.6	45.0	45.0
	Motivation cannot be applied in real life	1	1.1	5.0	50.0
	Motivation increases enthusiasm	1	1.1	5.0	55.0
	Professors can help to increase learning	1	1.1	5.0	60.0
	Motivation enhances learning	2	2.1	10.0	70.0
	Learning English spelling can also be of great help	1	1.1	5.0	75.0
	Motivation facilitates learning	2	2.1	10.0	85.0
	Motivation leads to improvement in learning	1	1.1	5.0	90.0
	Motivation can improve my social status	1	1.1	5.0	95.0
	It also depends on teaching methodology	1	1.1	5.0	100.0
	Total	20	21.3	100.0	

## Discussion

The purposes of this study are three-fold; this study intended to investigate the potential effects of different learner variables of age, major (field of study), and occupation on intrinsic and extrinsic motivation and to determine the possible relationship between intrinsic, extrinsic, and total motivation. It also sought to specify the themes which were related to students’ motivation. Using Multiple Analysis of Variance (MANOVA) and Pearson correlation, our study revealed that demographic variables such as age, field of study, and professional status did not have any influence on intrinsic or extrinsic motivation among selected learners. Nonetheless, we identified significant positive correlations between intrinsic motivation and the total motivation index and between extrinsic motivation and the total motivation index. Additionally, qualitative data collected from interviews underwent thematic analysis, which highlighted new and insightful trends based on the participants’ responses. The findings indicated that the majority of interviewees were driven by intrinsic motives and viewed motivation as a significant factor which could improve and facilitate learning.

### Discussion of quantitative findings

It came to light that age did not influence the total motivation index. A plausible explanation is that most of the participants were in the age range of 30–39 and 40–49 and a few students were above 50 or below 29. In samples in which half of the students are of young age and another half of middle or old age, there are greater odds for the significant effect on motivation. However, by chance, most of the participants who were involved in the present study were in the middle-age group. Additionally, our participants may have stable and similar levels of intrinsic motivation which have not been affect by the external factor of age. The fact that these learners experience the same cultural and educational norms and settings may also have led to close levels of motivation in different age groups. The finding of our study is in contrast with the inquiry which reported statistically significant differences between older and younger participants [43]. However, it is in the same line with [42]’s findings, indicating that the relationship between age and motivation was not significantly different.

In our study, the oldest participants (those who were in the age range of 50 and above) obtained the highest vectors of means in intrinsic motivation. The results of the qualitative studies also indicated that our participants were intrinsically motivated. Most of our students were middle-aged; therefore, our study supports the Socioemotional selectivity theory which stated that as people age, they move from extrinsic rewards to intrinsic motives and become more intrinsically motivated [36].

Age exerted no impact on intrinsic motivational orientation and this outcome was in contrast with the reports of some studies which showed that age influenced intrinsic motivation [14, 44, 45]. The findings are also in contrast with those of a study which maintained that age had some effects on extrinsic motivation [15]. Contrary to the results of our study, it was reported in some previous studies that age affected Irish participants’ intrinsic motivation [50] in that there was a positive relationship between age and intrinsic motivation.

It was also found that age did not impact extrinsic motivation; these findings are in the same line with those of some researchers who clarified that age had no effect on extrinsic motivation [45], while they are in contrast with the works of some other scholars who found that age had an impact on extrinsic motivation [39, 50]. Specifically, it was confirmed that a strong negative relationship existed between age and extrinsic motivation [50]. Furthermore, it was revealed that the age variable had no influence on intrinsic/extrinsic motivation. This result is in the same line with those of other scholars [46]. Interesting enough, the oldest students (those who were in the age range of 50 and above) had the highest intrinsic, extrinsic, and total motivation index. It can be explained by the fact that when a student starts learning at the age of 50 and above, he/she must be very motivated; this motivation may be a self-drive in him/her that tempts him/her to learn the forms of his/her intrinsic motivational behavior or probably a tendency to pursue learning for obtaining external rewards, praise, and good grades which shapes her extrinsic motivation. In another study, some similar findings were reported as older participants had different and high intrinsic motivation indices because the separate extrinsic motivation indices in their study decreased with age [51]. The aforementioned participants were found to be extrinsically motivated at earlier stages in their life, and as they aged, they became disinterested in extrinsic motivators. It was reported that there was a statistically significant effect of age on Iranian EFL learners’ motivation level [49] and different types of motivation were associated with age [52]. Concerning the age and major variables, our study challenged the findings of previous studies; most previous studies showed that age and area of study influenced the participants’ intrinsic/extrinsic motivation. However, our findings are in the same line with those of a research study [51] which illustrated that older learners had high intrinsic motivation indices.

The participants’ major did not affect intrinsic/extrinsic motivation. The lack of effect of major on intrinsic/extrinsic motivation may be attributed to various factors. We may set forth that in the culture in which our study was conducted, the effect of students’ long-term goals and cultural norms may be more conspicuous and exert greater impacts on their motivation than students’ fields of study. Therefore, instructors should strive to motivate their students based on the cultural norms which are more prevalent. Moreover, participants were all students of fields of humanities, and different results might be obtained if cross-comparisons were made between students of different areas of knowledge such as natural sciences and engineering. The results of our study were not in the same line with other authors’ findings which delineated that participants at different education programs such as elementary education, secondary education, early childhood education, educational research and evaluation, sciences education, Master of Education (M.Ed) and Bachelor of Education (B.Ed) differed in light of motivational aspect [47]. Although students of Theology and the Arabic language obtained the highest vectors of means in total motivation, their means were very close to those of students of other majors (Elementary Education, Educational Affairs and Social studies) and the differences were not statistically significant.

It was also found, in our study, that occupation did not affect intrinsic/extrinsic motivation. Given the fact that most of the participants were teachers or education practitioners, great occupational variety could not be observed among them. If participants were engaged in basically different professions, for example some of them were nurses, some engineers and some teachers, there was a greater chance that their professional status affected the motivational orientation. The participants were all school educators who were either elementary school teacher, secondary school teachers or assistant for educational affairs in schools. Therefore, their work is similar in its very educational nature and the participants who are all working in the same province, studying at the same university (the university is a teacher-training one), experiencing the same supportive environment and undergoing the same professional and academic concerns, may have shared professional values and similar motivational goals. Therefore, these participants were not significantly different in their intrinsic and extrinsic motivation.

Our result was in line with the reports of a study which revealed that workplace did not impact extrinsic motivational factors [48] and tied with the investigation which showed that extrinsic work motivation did not influence work engagement [57] though it was in contrast with the research which clarified that there was a positive relationship between intrinsic motivation and employee engagement, and between extrinsic motivation and employee engagement [58]. Our findings also differed from those of the study which indicated that different types of motivations had a positive association with work engagement [52]. Moreover, our findings were inconsistent with the research which reported that intrinsic motivation had a significant effect on nurses’ work engagement [57]. Elementary school teacher, secondary school teachers, and assistants for educational affairs obtained very close vectors of means, and their occupation did not have significant impacts on their motivation. This could be attributed to the fact that these jobs were intrinsically similar in nature and did not yield vast differences.

Pearson correlation results showed that there was a non-significant relationship between intrinsic and extrinsic motivation. This may be caused by the effect of some personality traits and environmental factors. Provided that our study is replicated in the future with more diverse sample sizes, different results may be obtained. Our finding is in contrast with the findings of a study which was carried out in a college in Karachi and found a significant relationship between the two types of motivation [67] and with the study which accentuated that there was a positive correlation between intrinsic and extrinsic motivation [68] and the investigation which indicated that intrinsic and extrinsic motivation could coexist and were not contradictory [69]. That intrinsic and extrinsic types of motivation were not related in our study might be due to the fact that the two forgoing motivation types were potentially separate constructs.

#### The final conceptual framework

**Fig. 6:**
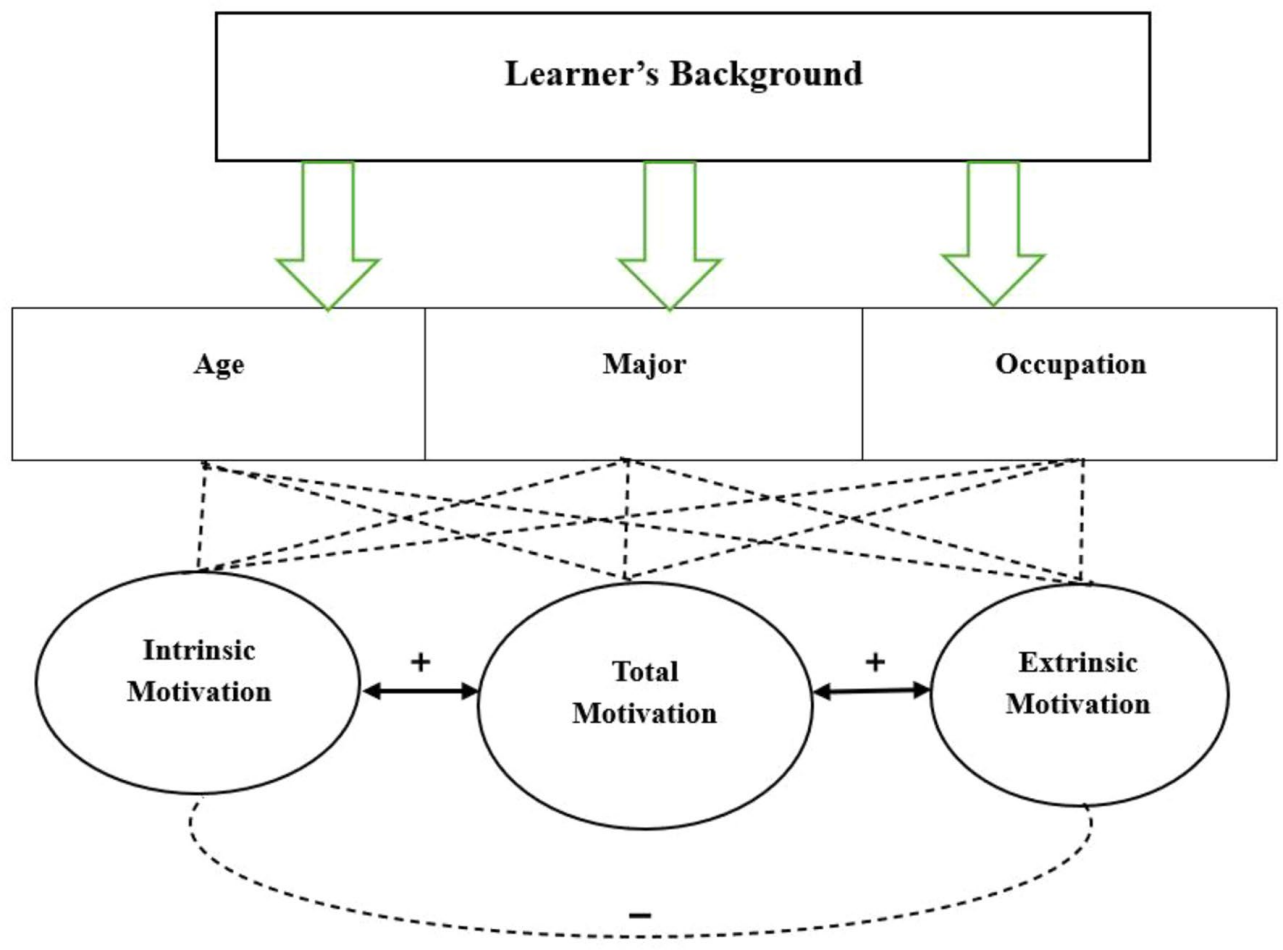
Final conceptual model of factors affecting intrinsic/extrinsic motivation

Our primary conceptual framework was developed into a final all-inclusive framework according to the results which were obtained in our study. Double-headed arrows show that there are reciprocal relationships between variables and plus sign shows that there is a positive relationship between variables; for example, there are positive relationships between intrinsic motivation and total motivation and between extrinsic motivation and total motivation. However, a negative sign clarifies that there is a negative relationship between variables, as is the case with variables intrinsic and extrinsic motivation. Moreover, dotted lines indicate that there exists no relationship among the factors. Thus, the effects of the background variables of age, major, and occupation on intrinsic, extrinsic, and total motivation are illustrated by dotted lines. Our study findings are consistent with those of a scholarly work which reported that the demographic factors of age and educational qualifications had no impact on either intrinsic or extrinsic motivation [70] and inconsistent with a study in which profession was shown to exert a significant influence on participants’ intrinsic and extrinsic motivation [71]. The final conceptual framework of the study is shown in Fig. 6 below.

### Discussion of qualitative findings

Employing Attride-Stirling’s (2001) thematic network technique, which is a rigorous approach, we analyzed the collected qualitative data. We transcribed the interview data and developed a coding framework that consisted of codes representing main concepts. In the initial coding phase, the transcripts were broken down into meaningful segments, and these segments were annotated with relevant codes, thereby extracting the lowest-order premises. Once the basic themes were identified, they were organized into organizing themes which facilitated the comprehension of how different themes were related. Then, the most significant global themes were derived from the organizing themes.

Excel was used for creating visual representations and thematic networks which demonstrated the connections between different themes. Its user-friendly design paved the way for data manipulation and graphical representation. We used SPSS version 23 to quantitatively support the qualitative findings. For example, the frequency of responses of each theme was quantified and commonly mentioned responses were deemed more significant, guiding the focus on the themes that appeared consistently across interviews and allowing for comparative analysis about predominant types of motivation among students.

The results of qualitative interviews showed that 22% of the responses to the main interview question which sought the reasons why they learned new material were either related to their tendency to receive praise or to get better grades which both could be ascribed to extrinsic motivation; in addition, 78% of the responses were related to intrinsic motivation. Therefore, the selected students are more driven by intrinsic motives. The same result was obtained through the analysis of quantitative data when we compared the mean scores of intrinsic motivation with extrinsic motivation among all age groups, majors and professional status as students obtained higher means in intrinsic motivation. Therefore, educators had better tailor their instructional strategies to better engage the students based on their individual characteristics. They can focus on their students’ personal growth, deep learning, critical thinking and self-directed learning. Educators can also offer topics which are personally meaningful to students and help them use their learning in real-world contexts.

Interviewees in this study maintained that they learned new materials because they were eager to expand their knowledge. This maybe due to the fact that most of these students were intrinsically motivated, as revealed from the results of the interviews. This finding aligns with that of a study which indicated that EFL learners were highly intrinsically motivated [60]. Likewise, it has been emphasized that intrinsically motivated individuals like to increase their knowledge and are self-motivated in pursuit of knowledge, however, extrinsic drive should find a reason to pursue knowledge [72].

When interviewees’ motives were probed for learning new materials, it was found that they did believe that learning was essential and that it was the best incentive, and they were eager to generalize what they had learned to broader contexts. Moreover, when the relationship between motivation and learning was scrutinized, it was found that for interviewees in this study, motivation was a construct of great importance which could not only enhance learning but could also facilitate the language learning process.

### Limitations

There are several limitations in this study as to the participants. First, all the students in this study were bachelor’s degree students. Second, this work was confined to students who were non-native speakers whose mother tongues were not English. Third, as all the students at the selected university were female students who were prospective teachers, the study was limited to female participants. Fourth, as cluster sampling was used, undergraduate students who were expected to participate in the research survey and the interviews were divided into clusters and random samples of these clusters were selected. Fifth, all the students were being trained to be teachers or to be employed in teaching sectors. Therefore, there was not great diversity in profession. Sixed, the university was not a thickly populated one.

In the university in which the study was carried out, no doctoral degree students were studying. Master’s students were also a few in number. Moreover, the study was carried out in an EFL environment and we had no access to students studying in an ESL context. The university had no male students; therefore, we had to perform the study on female students. Cluster sampling was used since it is practical approach to research on students who are prospective teachers. These students were studying in a university the entrance to which needs excellent scores at the National Entrance Exam as many in Iran would like to enter this university since from the first day of entrance to the university, these students are considered employed and receive monthly salaries. If other studies are replicated with different levels of study, contexts of learning, gender, sampling procedures, professional status and sample size, some significant results might be obtained regarding some of the background variables.

### Theoretical implications

Up to now, many studies on intrinsic/extrinsic motivation have focused on the motivational orientations of students. Knowing the factors which influence the motivation of learners can be illuminating; however, even the studies which explored the effect of background variables, such as occupational influence, were mostly implemented in participants’ workplace. Studies in which the aforementioned factors are investigated among learners in different educational settings are few in number. Such studies should be done by teachers who are in direct contact with the learners; however, as an expert highlighted [73] since teachers are more involved in teaching and classroom management, they are not eager to do research on such studies.

In our study, the factors of age, major and occupation did not impact motivation. Therefore, the idea that motivation is not a static trait rather it is a dynamic process, as has been put by some scholars [74] is supported in our study considering motivation as a universal, complex and multifaceted trait which is less affected by external factors since it is affected by various factors which are beyond demographic and occupational characteristics [75]. It also supports the theories of self-determination which contends that motivation is driven by internal factors [20] rather than external factors such as age, major or occupation and is in line with the theory of intrinsic motivation which states that people are less motivated by external factors [20]. It is also necessary to reassess the theories and models which may suggest that factors such as age, major and occupation play a role in university students’ intrinsic/extrinsic motivation in an EFL context and to examine other factors which may increase university students’ motivation.

### Pedagogical implications

When practitioners of language teaching know about the variables which are of paramount importance in enhancing the students’ motivation, their core competency is formed and that helps them take their further steps in accordance with this precious knowledge. In our study, it was revealed that the participants are more intrinsically motivated. As these students are more guided by their own interest, instructors can provide a supportive learning environment in which students can exercise learner autonomy and make their own learning decisions and educators can offer intellectually-stimulating homework in which students foster their critical thinking skills and elevate their academic competencies.

### Contributions and suggestions

The current study expands the existing understanding of the area of motivation in general and intrinsic/extrinsic motivation in particular. What is of paramount importance is for instructors to pay sufficient attention to their learners’ individual differences so that they can cater to their learners’ diverse educational needs as “learning processes vary from person to person and the awareness of the fact that dissimilarity exists among learners’ preferences in learning, determines teachers to accommodate the learners’ needs accordingly” [55 p70]. In addition, supplying tasks and teaching methodologies that benefit different types of learners with different features contributes to practical learning [56].

In our study, that the variables of age, major and occupation had no effects on the two motivation types can challenge the previous assumptions and calls for in-depth investigations of factors which may influence intrinsic/extrinsic motivation in different learning environments. Our research findings show experts in the field that the construct of motivation is more complex than it initially looked and more focused interventions are needed to be followed to obtain better educational outcomes. This study was carried out in a context in which learners learned English as a foreign language. If the effects of the same factors are investigated in a context in which English is a second language (ESL), different results may be obtained about the impact of learner background variables on different types of motivation. Moreover, the university in which the study was implemented was a teacher-training university. As most of them were shown to be more intrinsically motivated, it is advisable that they be provided with professional development programs. These future generation of teachers will be autonomous, growth-oriented and can set valuable goals for their learners.

There are different topics which are pertinent to motivation which warrant studying. One interesting topic is the replication of our study in an ESL context. Similar studies can be done at a a larger scale in different cultures and countries. To understand the role of motivation and the factors that drive student engagement at universities, studies can be done on the relationships among different personality traits and motivation and various environmental factors and motivational types. The relationship between sensory preferences and motivational orientation can open up new avenues in psychological and education research. Another revealing topic is the effect of Teachers’ Motivational Strategies (TMSs) on students’ intrinsic and extrinsic motivation. Bringing real-life situations to classrooms is one of the demanding missions for instructors and practitioners of language teaching. Teachers and instructors are suggested to perform a need analysis at the beginning of each semester and try to attune their lesson plans to the needs of their students.

## Conclusion

This study aimed to investigate the factors which might affect intrinsic and extrinsic motivation. Thus, effects of the learner background variables of age, major, and occupation were explored on intrinsic/extrinsic motivation in a sample of a public university students in an EFL context. Moreover, the possible relationships among intrinsic extrinsic and total motivation were examined and the motivational themes which were revealed from interviews were appraised. Furthermore, we strived to bridge existing gaps in the literature regarding the interplay of various factors affecting motivation in educational settings, particularly in EFL contexts. Our results indicated that these demographic factors did not have a significant influence on either type of motivation. However, we found positive correlations between intrinsic motivation and the overall motivation index and between extrinsic motivation and the overall motivation index. Qualitative interviews revealed a clear preference for intrinsic motivation among participants, many of whom regarded it as vital for improving their learning experiences.

It has also been illuminated that motivation is a dynamic trait which is less influenced by external factors. In the EFL context in which our study was performed, instructors can follow more homogeneous classroom motivational techniques and strategies while teaching different courses in general and English courses in particular since neither type of motivation was influenced by the variables of major, age and occupation. Furthermore, some enlightening trends were found in the interviewees’ responses in connection with the effects of motivation in learning; also, it was elucidated that the selected interviewees followed intrinsic motives more than extrinsic ones, and to them motivation was a construct of overriding importance which led to greater learning. Therefore, instructors can benefit from some strategies to boost their students’ intrinsic motivation by empowering students with the idea of a conscious choice, setting higher goals, following self-motivating strategies, providing autonomy-supportive environments, reinventing the system of rewards, and making students feel education is a choice not an indispensable requirement or a necessity.

The age variable did not influence the learners’ intrinsic/extrinsic motivation as most of the participants were middle-aged. Likewise, the variable of major did not have any effects on motivation since most students’ fields were related to educational and social sciences which have similarities in nature. Thus, instructors can follow more similar classroom motivational techniques and strategies. Given that participants were engaged in basically different professions, there were greater odds that their professional status affected their motivational orientation. However, that was not the case in the present study since all participants were either teachers or education practitioners among whom profession did not exert influences on intrinsic/extrinsic motivation. Therefore, their instructors do not have to vary class activities in line with students’ profession. Our participants were female Bachelor’s degree students studying in an EFL context who were training to be teachers. This study can be replicated at universities following coed education systems with different educational levels both in EFL and ESL contexts to make comparisons and contrasts among different groups of learners. On the whole, since an increase in motivation would impel language learners to make the most of available resources to succeed in their learning efforts, instructors should attempt to design some motivational strategies for classroom application and apply motivational techniques to obtain most fruitful results in different educational settings.

## Electronic supplementary material

Below is the link to the electronic supplementary material.

Supplementary Material 1

## Data Availability

The author confirms that all data generated or analyzed during this study are included in this published article.
