# Supplementary material for: Optimizing EFL learning: exploring the role of learner background factors and the nuances of their effects on intrinsic and extrinsic motivation among university students in a mixed-methods study
Source: BMC Psychol. 2024 Oct 5;12:535. doi: 10.1186/s40359-024-02034-8 (PMC11453079; doi:10.1186/s40359-024-02034-8)
Supplement: Supplementary file 1 — Supplementary Material 1 [file 40359_2024_2034_MOESM1_ESM.docx]

**Appendix**

***Tables***

**Table A.1** Students’ Responses to “Why Do You Learn New Materials?”

|  | | Frequency | Percent | Valid Percent | Cumulative Percent |
| --- | --- | --- | --- | --- | --- |
| Valid | For more satisfaction | 1 | 1.1 | 5.0 | 5.0 |
|  | For more effectiveness | 1 | 1.1 | 5.0 | 10.0 |
|  | Because I am interested | 1 | 1.1 | 5.0 | 15.0 |
|  | Because I want to learn more lexical items | 1 | 1.1 | 5.0 | 20.0 |
|  | I want to expand my knowledge | 5 | 5.3 | 25.0 | 45.0 |
|  | I don't plan to be praised by others | 1 | 1.1 | 5.0 | 50.0 |
|  | I plan to review my lessons | 1 | 1.1 | 5.0 | 55.0 |
|  | For better grades | 3 | 3.2 | 15.0 | 70.0 |
|  | Because I want to teach others | 1 | 1.1 | 5.0 | 75.0 |
|  | For effective communication | 1 | 1.1 | 5.0 | 80.0 |
|  | For a better life | 1 | 1.1 | 5.0 | 85.0 |
|  | I like to be praised by others | 1 | 1.1 | 5.0 | 90.0 |
|  | For more success | 1 | 1.1 | 5.0 | 95.0 |
|  | I like to apply different learning methods | 1 | 1.1 | 5.0 | 100.0 |
|  | Total | 20 | 21.3 | 100.0 |  |

**Table A.2** Students’ Responses to “Do You Learn New Materials for the Sake of Learning?”

|  | | Frequency | | Percent | | Valid Percent | Cumulative Percent | |
| --- | --- | --- | --- | --- | --- | --- | --- | --- |
| Valid | Learning is necessary | | 5 | | 5.3 | 25.0 | 25.0 |  |
|  | Learning is the best incentive | | 5 | | 5.3 | 25.0 | 50.0 |  |
|  | I don't have a definite plan for learning | | 1 | | 1.1 | 5.0 | 55.0 |  |
|  | I try to learn new lexical items | | 1 | | 1.1 | 5.0 | 60.0 |  |
|  | I like to generalize what I have learned to broader contexts | | 3 | | 3.2 | 15.0 | 75.0 |  |
|  | I only try to learn materials related to my university courses | | 1 | | 1.1 | 5.0 | 80.0 |  |
|  | I have to learn and apply the learned material | | 1 | | 1.1 | 5.0 | 85.0 |  |
|  | I learn new materials to get better scores | | 1 | | 1.1 | 5.0 | 90.0 |  |
|  | Learning helps me keep new materials in my memory | | 1 | | 1.1 | 5.0 | 95.0 |  |
|  | I try to expand my knowledge through learning | | 1 | | 1.1 | 5.0 | 100.0 |  |
|  | Total | | 20 | | 21.3 | 100.0 |  |  |
| Missing | System | | 74 | | 78.7 |  |  |  |
| Total | | | 94 | | 100.0 |  |  |  |
